# Supplementary material for: Translation and Cross-Cultural Adaptation of the Pediatric Outcomes Data Collection Instrument into the Italian Language
Source: Children (Basel). 2022 Jun 8;9(6):853. doi: 10.3390/children9060853 (PMC9221952; doi:10.3390/children9060853)
Supplement: Supplementary file 1 [file children-09-00853-s001.zip › Supplementary Annex 1.pdf]

Supplementary Annex 1. PODCI Italian versions

Questionario per

Adolescenti (auto compilato)

Creato da:

American Academy of Orthopaedic Surgeons®

Pediatric Orthopaedic Society of North America

American Academy of Pediatrics

Shriner's Hospitals

Deve essere compilato dagli adolescenti tra gli 11 – 18 anni

Basato sulla Versione 2.0 del Pediatrics–Parent/ Adolescent Outcomes Intrument

Conosciuto anche come PODCI ("Pediatric Outcomes Data Collection Instrument")

Valutazione della Salute degli Adolescenti (auto compilato)

Data di oggi / /

Grazie per aver completato questo questionario!

Questo questionario ci aiuterà a comprendere meglio il tuo stato di salute generale e gli eventuali problemi legati alle condizioni ossee e muscolari.

La compilazione di questo questionario è completamente volontaria e le tue risposte saranno mantenute con la massima riservatezza.

Per favore, rispondi ad ogni domanda. Alcune domande possono assomigliare ad altre, ma ognuna è diversa.

Non ci sono risposte giuste o sbagliate. Se non sei sicuro di come rispondere a una domanda, dai solo la migliore risposta possibile. Puoi inserire commenti a margine. Leggeremo tutti i tuoi commenti, quindi sentiti libero di farne quanti ne desideri.

Data di nascita del paziente / /

Valutazione della Salute degli Adolescenti (auto compilato)

Alcuni problemi possono rendere difficile lo svolgimento di molte attività, come mangiare, fare il bagno, fare i compiti scolastici e giocare con gli amici. Vorremmo scoprire come stai. (Cerchia una risposta su ogni riga.)

Durante **l'ultima settimana** quanto è stato difficile per te:

		**Facile**	**Un po' difficile**	**Molto difficile**	**Non ci sono riuscito**
**1.**	Sollevare libri pesanti?	1	2	3	4
**2.**	Versare un litro di latte?	1	2	3	4
**3.**	Aprire un barattolo precedentemente aperto?	1	2	3	4
**4.**	Usare una forchetta o un cucchiaio?	1	2	3	4
**5.**	Pettinarti?	1	2	3	4
**6.**	Abbottonare una camicia?	1	2	3	4
**7.**	Indossare una giacca?	1	2	3	4
**8.**	Scrivere con una penna?	1	2	3	4

9. In media, **negli ultimi 12 mesi**, quanto spesso hai saltato la scuola per motivi di salute?

1. Raramente
2. Una volta al mese
3. Due o tre volte al mese
4. Una volta a settimana
5. Più di una volta a settimana
6. Non vado a scuola

Durante **l'ultima settimana** quanto ti sei sentito a tuo agio con: (Cerchia la Tua risposta ad ogni domanda.)

		**Molto felice**	**Un po' felice**	**Insicuro**	**Un po' triste**	**Molto infelice**
**10.**	Il tuo aspetto?	1	2	3	4	5
**11.**	Il tuo corpo?	1	2	3	4	5
**12.**	I vestiti e le scarpe che indossi?	1	2	3	4	5
**13.**	La tua capacità di fare le stesse cose che fanno i tuoi amici?	1	2	3	4	5
**14.**	La Tua salute in generale?	1	2	3	4	5

Valutazione della Salute degli Adolescenti (auto compilato)

Durante **l'ultima settimana**, quanto tempo: (cerchia una risposta ad ogni domanda)

		La maggior parte delle volte	Qualche volta	Un po' di volte	Mai
**15.**	Ti sei sentito stanco?	1	2	3	4
**16.**	Ti sei sentito pieno di energia?	1	2	3	4
**17.**	Il dolore ha interferito con le tue attività?	1	2	3	4

Durante **l'ultima settimana**, è stato facile o difficile per te: (cerchia una risposta ad ogni domanda)

		Facile	Un po' difficile	Molto difficile	Non ci sono riuscito
**18.**	Correre per brevi distanze?	1	2	3	4
**19.**	Andare in bicicletta?	1	2	3	4
**20.**	Salire tre rampe di scale?	1	2	3	4
**21.**	Salire una rampa di scale?	1	2	3	4
**22.**	Camminare per più di un chilometro?	1	2	3	4
**23.**	Camminare per tre isolati?	1	2	3	4
**24.**	Camminare per un isolato?	1	2	3	4
**25.**	Salire e scendere dall'autobus?	1	2	3	4

26. Con che frequenza hai bisogno dell'aiuto di un'altra persona per camminare e/o arrampicarti? (Cerchia una risposta)

1 Mai   2 A volte   3 Circa la metà delle volte   4 Spesso   5 Sempre

27. Con che frequenza usi dispositivi di assistenza (come tutori, stampelle o sedia a rotelle) per camminare e/o arrampicarti? (Cerchia una risposta)

1 Mai   2 A volte   3 Circa la metà delle volte   4 Spesso   5 Sempre

Durante **l'ultima settimana**, è stato facile o difficile per te:(cerchia una risposta ad ogni domanda)

		Facile	Un po' difficile	Molto difficile	Non ci sono riuscito
**28.**	Stare in piedi per lavarti le mani e la faccia?	1	2	3	4
**29.**	Sedersi su una sedia normale senza tenersi?	1	2	3	4
**30.**	Salire e scendere dal gabinetto?	1	2	3	4
**31.**	Salire e scendere dal letto?	1	2	3	4
**32.**	Aprire la maniglia della porta?	1	2	3	4
**33.**	Piegarsi in avanti per raccogliere qualcosa da terra?	1	2	3	4

34. Con che frequenza hai bisogno dell'aiuto di un'altra persona per stare seduto o in piedi? (Cerchia una risposta)

1 Mai   2 A volte   3 Circa la metà delle volte   4 Spesso   5 Sempre

35. Con che frequenza utilizzi dispositivi di assistenza (come tutori, stampelle o sedia a rotelle) per stare seduto o in piedi? (Cerchia una risposta)

1 Mai   2 A volte   3 Circa la metà delle volte   4 Spesso   5 Sempre

36. Riesci partecipare ad **attività ricreative all'aperto** con altri bambini della stessa età? Ad esempio: ciclismo, pattinaggio, escursionismo, jogging... (Cerchia una risposta).

1 Si, facilmente   2 Si, ma è un po' difficile   3 Si, ma è molto difficile   4 No

Se hai risposto "no" alla domanda 36, la tua attività era limitata da: (Cerchia tutte le risposte pertinenti)

		**Si**
**37.**	Dolore?	1
**38.**	Il tuo livello di salute generale?	1
**39.**	Indicazioni del Medico o dei Genitori?	1
**40.**	Hai paura di non piacere agli altri?	1
**41.**	Non ti piacciono gli sport all'aperto?	1
**42.**	Attività fuori stagione?	1

43. Riesci a partecipare **a giochi o sport di squadra** con altri ragazzi della tua stessa età? (Ad esempio: basket, calcio, pallavolo, salto con la fune…) (Cerchia una risposta).

1 Si, facilmente   2 Si, ma è un po' difficile   3 Si, ma è molto difficile   4 No

Se hai risposto "no" alla domanda 43, la tua attività era limitata da: (Cerchia tutte le risposte pertinenti)

		**Si**
**44.**	Dolore?	1
**45.**	Il tuo livello di salute generale?	1
**46.**	Indicazioni del Medico o dei Genitori?	1
**47.**	Hai paura di non piacere agli altri?	1
**48.**	Non ti piacciono gli sport di squadra?	1
**49.**	Attività fuori stagione?	1

Valutazione della Salute degli Adolescenti (auto compilato)

50. Riesci a partecipare **a sport di livello agonistico** con altri ragazzi della stessa età?
(Ad esempio: hockey, basket, calcio, baseball, nuoto, corsa [pista o sci di fondo], ginnastica o danza)
(Cerchia una risposta).

1 Si, facilmente 2 Si, ma è un po' difficile 3 Si, ma è molto difficile 4 No

Se hai risposto "no" alla domanda 50, la tua attività era limitata da: (Cerchia tutte le risposte pertinenti)

	Si
**51.** Dolore?	1
**52.** Il tuo livello di salute generale?	1
**53.** Indicazioni del Medico o dei Genitori?	1
**54.** Hai paura di non piacere agli altri?	1
**55.** Non ti piacciono gli sport a livello agonistico?	1
**56.** Attività fuori stagione?	1

57. Quante volte **nell'ultima settimana** ti sei incontrato per fare attività con i tuoi amici? (cerchia una risposta)

1 Spesso 2 A volte 3 Raramente o Mai

Se hai risposto "A volte" o "Raramente o Mai" alla domanda 57, la tua attività era limitata da:
(Cerchia tutte le risposte pertinenti)

	Si
**58.** Dolore?	1
**59.** Il tuo livello di salute generale?	1
**60.** Indicazioni del Medico o dei Genitori?	1
**61.** Hai paura di non piacere agli altri?	1
**62.** Gli amici non erano disponibili?	1

63. Quante volte **nell'ultima settimana** hai partecipato alla **ginnastica scolastica/ricreazione**?

1 Spesso 2 A Volte 3 Mai o Raramente 4 Non abbiamo ore di ginnastica

Se hai risposto "A Volte" o "Mai o Raramente" alla domanda 63 sopra, la tua attività era limitata da:
(Cerchia tutte le risposte pertinenti)

	Si
**64.** Dolore?	1
**65.** Il tuo livello di salute generale?	1
**66.** Indicazioni del Medico o dei Genitori?	1
**67.** Hai paura di non piacere agli altri?	1
**68.** Non ti piace fare ginnastica a scuola?	1
**69.** Non siamo in periodo scolastico?	1
**70.** Non frequento la scuola	1

71. È facile o difficile per te fare amicizia con ragazzi della tua stessa età? (Cerchia una risposta)

1 Generalmente Facile 2 Spesso Facile 3 Spesso Difficile 4 Generalmente Difficile

72. Quanto dolore hai avuto durante **l'ultima settimana**? (Cerchia una risposta)

1. Nessuno 2. Molto Poco 3. Lieve 4. Moderato 5. Severo 6. Molto severo

73. Durante **l'ultima settimana**, quanto il dolore ha interferito con le tue normali attività (comprese quelle a casa, fuori casa e a scuola)? (Cerchia una risposta)

1. Per Nulla 2. Un po' 3. Moderatamente 4. Un bel po' 5. Estremamente

Quali aspettative hai circa il tuo trattamento?
Come risultato del mio trattamento, mi aspetto: (Cerchia una risposta su ogni riga.)

		Sicuramente si	Probabilmente si	Non sono sicuro	Probabilmente no	Sicuramente no
**74.**	Avere meno dolore	1	2	3	4	5
**75.**	Avere un aspetto migliore	1	2	3	4	5
**76.**	Sentirmi meglio con me stesso	1	2	3	4	5
**77.**	Dormire più comodamente	1	2	3	4	5
**78.**	Essere in grado di svolgere attività in casa	1	2	3	4	5
**79.**	Poter fare di più a scuola	1	2	3	4	5
**80.**	Essere in grado di giocare di più con gli amici.	1	2	3	4	5
**81.**	Poter fare più sport	1	2	3	4	5
**82.**	Non avere dolore e limitazione da adulto	1	2	3	4	5

83. Se dovessi passare il resto della tua vita con le tue condizioni ossee e muscolari **come sono adesso**, come ti sentiresti al riguardo? (Cerchia una risposta)

1. Molto soddisfatto
2. Più o meno soddisfatto
3. Né soddisfatto, né insoddisfatto
4. Più o meno insoddisfatto
5. Molto insoddisfatto

Questionario per

Adolescenti (compilato dai genitori)

Creato da:

American Academy of Orthopaedic Surgeons®

Pediatric Orthopaedic Society of North America

American Academy of Pediatrics

Shriner's Hospitals

Deve essere compilato dai genitori del ragazzo tra gli 11 – 18 anni

Basato sulla Versione 2.0 del Pediatrics–Parent/ Adolescent Outcomes Intrument

Conosciuto anche come PODCI ("Pediatric Outcomes Data Collection Instrument")

Valutazione della Salute degli Adolescenti (compilato dai genitori)

Data di oggi / /

Grazie per aver completato questo questionario!

Questo questionario ci aiuterà a comprendere meglio lo stato generale di salute di Suo/a figlio e gli eventuali problemi legati alle condizioni ossee e muscolari.

La compilazione di questo questionario è completamente volontaria e le Sue risposte saranno mantenute con la massima riservatezza.

Per favore risponda ad ogni domanda. Alcune domande possono assomigliare ad altre, ma ognuna è diversa.

Non ci sono risposte giuste o sbagliate. Se non è sicuro di come rispondere a una domanda, dia solo la migliore risposta possibile. Può inserire commenti a margine. Leggeremo tutti i tuoi commenti, quindi sentiti libero di farne quanti ne desideri.

Data di nascita del paziente / /

Valutazione della Salute degli Adolescenti (compilato dai genitori)

Alcuni problemi possono rendere difficile lo svolgimento di molte attività, come mangiare, fare il bagno, fare i compiti scolastici e giocare con gli amici. Vorremmo scoprire come sta Suo/a/a figlio/a.
(Cerchia una risposta su ogni riga.)

Durante **l'ultima settimana** quanto è stato difficile per Suo/a figlio/a:

		Facile	Un po' difficile	Molto difficile	Non ci sono riuscito
**1.**	Sollevare libri pesanti?	1	2	3	4
**2.**	Versare un litro di latte?	1	2	3	4
**3.**	Aprire un barattolo precedentemente aperto?	1	2	3	4
**4.**	Usare una forchetta o un cucchiaio?	1	2	3	4
**5.**	Pettinarsi?	1	2	3	4
**6.**	Abbottonare una camicia?	1	2	3	4
**7.**	Indossare una giacca?	1	2	3	4
**8.**	Scrivere con una penna?	1	2	3	4

9. In media, **negli ultimi 12 mesi**, quanto spesso Suo/a figlio/a ha saltato la scuola per motivi di salute?

1. Raramente
2. Una volta al mese
3. Due o tre volte al mese
4. Una volta a settimana
5. Più di una volta a settimana
6. Non va a scuola

Durante **l'ultima settimana** quanto Suo/a figlio/a si è sentito a tuo agio con: (Cerchia una risposta per ogni domanda.)

		Molto felice	Un po' felice	Insicuro	Un po' triste	Molto infelice
**10.**	Il Suo aspetto?	1	2	3	4	5
**11.**	Il Suo corpo?	1	2	3	4	5
**12.**	I vestiti e le scarpe che indossa?	1	2	3	4	5
**13.**	La Sua capacità di fare le stesse cose che fanno i Suoi amici?	1	2	3	4	5
**14.**	La Sua salute in generale?	1	2	3	4	5

Valutazione della Salute degli Adolescenti (compilato dai genitori)

Durante **l'ultima settimana**, quanto tempo: (cerchia una risposta ad ogni domanda)

		La maggior parte delle volte	Qualche volta	Un po' di volte	Mai
**15.**	Suo/a figlio/a si è sentito stanco?	1	2	3	4
**16.**	Suo/a figlio/a si è sentito pieno di energia?	1	2	3	4
**17.**	Il dolore ha interferito con le attività di Suo/a figlio/a?	1	2	3	4

Durante **l'ultima settimana**, è stato facile o difficile per Suo/a figlio/a: (cerchia una risposta ad ogni domanda)

		Facile	Un po' difficile	Molto difficile	Non ci sono riuscito
**18.**	Correre per brevi distanze?	1	2	3	4
**19.**	Andare in bicicletta?	1	2	3	4
**20.**	Salire tre rampe di scale?	1	2	3	4
**21.**	Salire una rampa di scale?	1	2	3	4
**22.**	Camminare per più di un chilometro?	1	2	3	4
**23.**	Camminare per tre isolati?	1	2	3	4
**24.**	Camminare per un isolato?	1	2	3	4
**25.**	Salire e scendere dall'autobus?	1	2	3	4

26. Con che frequenza Suo/a figlio/a ha bisogno dell'aiuto di un'altra persona per camminare e/o arrampicarsi? (Cerchia una risposta)

1 Mai 2 A volte 3 Circa la metà delle volte 4 Spesso 5 Sempre

27. Con che frequenza Suo/a figlio/a usa dispositivi di assistenza (tutori, stampelle o sedia a rotelle) per camminare e/o arrampicarsi? (Cerchia una risposta)

1 Mai 2 A volte 3 Circa la metà delle volte 4 Spesso 5 Sempre

Durante **l'ultima settimana**, quanto è stato facile per Suo/a figlio/a:(cerchia una risposta ad ogni domanda)

		Facile	Un po' difficile	Molto difficile	Non ci sono riuscito
**28.**	Stare in piedi per lavarsi le mani e la faccia?	1	2	3	4
**29.**	Sedersi su una sedia normale senza tenersi?	1	2	3	4
**30.**	Salire e scendere dal gabinetto?	1	2	3	4
**31.**	Salire e scendere dal letto?	1	2	3	4
**32.**	Aprire la maniglia della porta?	1	2	3	4
**33.**	Piegarsi in avanti per raccogliere qualcosa da terra?	1	2	3	4

34. Con che frequenza Suo/a figlio/a ha bisogno dell'aiuto di un'altra persona per stare seduto o in piedi? (Cerchia una risposta)

1 Mai 2 A volte 3 Circa la metà delle volte 4 Spesso 5 Sempre

35. Con che frequenza Suo/a figlio/a utilizza dispositivi di assistenza (come tutori, stampelle o sedia a rotelle) per stare seduto o in piedi? (Cerchia una risposta)

1 Mai 2 A volte 3 Circa la metà delle volte 4 Spesso 5 Sempre

36. Riesce Suo/a figlio/a a partecipare ad **attività ricreative all'aperto** con altri bambini della sua stessa età? Ad esempio: ciclismo, pattinaggio, escursionismo, jogging... (Cerchia una risposta).

1 Si, facilmente 2 Si, ma è un po' difficile 3 Si, ma è molto difficile 4 No

Se ha risposto "no" alla domanda 36, l'attività di Suo/a figlio/a era limitata da: (Cerchia sì a tutte le risposte pertinenti)

		**Si**
**37.**	Dolore?	1
**38.**	Il Suo livello di salute generale?	1
**39.**	Indicazioni del Medico o dei Genitori?	1
**40.**	Ha paura che Suo/a figlio/a non piaccia agli altri?	1
**41.**	A Suo/a figlio/a non piacciono gli sport all'aperto?	1
**42.**	Reputa Suo/a figlio/a troppo piccolo?	1
**43.**	Attività fuori stagione?	1

44. Riesce Suo/a figlio/a a partecipare in **giochi o sport di squadra** con altri ragazzi della sua stessa età? (Ad esempio: basket, calcio, pallavolo, salto con la fune…) (Cerchia una risposta).

1 Si, facilmente 2 Si, ma è un po' difficile 3 Si, ma è molto difficile 4 No

Se ha risposto "no" alla domanda 43, l'attività di Suo/a figlio/a era limitata da: (Cerchia tutte le risposte pertinenti)

		**Si**
**45.**	Dolore?	1
**46.**	Il Suo/a livello di salute generale?	1
**47.**	Indicazioni del Medico o dei Genitori?	1
**48.**	Ha paura che Suo/a figlio/a non piaccia agli altri?	1
**49.**	A Suo/a figlio/a non piacciono gli sport di squadra?	1
**50.**	Reputa Suo/a figlio/a troppo piccolo?	1
**51.**	Attività fuori stagione?	1

Valutazione della Salute degli Adolescenti (compilato dai genitori)

52. Riesce Suo/a figlio/a a partecipare **a sport di livello agonistico** con altri ragazzi della sua stessa età? (Ad esempio: hockey, basket, calcio, baseball, nuoto, corsa [pista o sci di fondo], ginnastica o danza) (Cerchia una risposta).

1 Si, facilmente 2 Si, ma è un po' difficile 3 Si, ma è molto difficile 4 No

Se ha risposto "no" alla domanda 50, la Sua attività era limitata da: (Cerchia tutte le risposte pertinenti)

		Si
**53.**	Dolore?	1
**54.**	Il Suo livello di salute generale?	1
**55.**	Indicazioni del Medico o dei Genitori?	1
**56.**	Ha paura che Suo/a figlio/a non piaccia agli altri?	1
**57.**	A Suo/a figlio/a non piacciono gli sport a livello agonistico?	1
**58.**	Reputa Suo/a figlio/a troppo piccolo?	1
**59.**	Attività fuori stagione?	1

60. Quante volte **<u>nell'ultima settimana</u>** Suo/a figlio/a si è incontrato con i Suoi amici per fare attività? (cerchia una risposta)

1 Spesso 2 A volte 3 Raramente o Mai

Se ha risposto "A volte" o "Raramente o Mai" alla domanda 57, l'attività di Suo/a figlio/a era limitata da: (Cerchia tutte le risposte pertinenti)

		Si
**61.**	Dolore?	1
**62.**	Il Suo livello di salute generale?	1
**63.**	Indicazioni del Medico o dei Genitori?	1
**64.**	Ha paura che Suo/a figlio/a non piaccia agli altri?	1
**65.**	Gli amici non erano disponibili?	1

66. Quante volte **nell'ultima settimana** Suo/a figlio/a ha partecipato alla **ginnastica scolastica/ricreazione**?

1 Spesso 2 A Volte 3 Mai o Raramente 4 Non abbiamo ore di ginnastica/ricreazione

Se ha risposto "A Volte" o "Mai o Raramente" alla domanda 66, l'attività di Suo/a figlio/a era limitata da: (Cerchia tutte le risposte pertinenti)

		Si
**67.**	Dolore?	1
**68.**	Il Suo livello di salute generale?	1
**69.**	Indicazioni del Medico o dei Genitori?	1
**70.**	Ha paura che Suo/a figlio/a non piaccia agli altri?	1
**71.**	A Suo/a figlio/a non ti piace fare ginnastica a scuola?	1
**72.**	Non siamo in periodo scolastico?	1
**73.**	Suo/a figlio/a non frequenta la scuola	1

74. È facile o difficile per Suo/a figlio/a fare amicizia con ragazzi della sua età? (Cerchia una risposta)

1 Generalmente Facile 2 Spesso Facile 3 Spesso Difficile 4 Generalmente Difficile

Valutazione della Salute degli Adolescenti (compilato dai genitori)

75. Quanto dolore ha avuto Suo/a figlio/a durante **l'ultima settimana**? (Cerchia una risposta)

1. Nessuno 2. Molto Poco 3. Lieve 4. Moderato 5. Severo 6. Molto severo

76. Durante **l'ultima settimana**, quanto il dolore ha interferito con le normali attività di Suo/a figlio/a (comprese quelle a casa, fuori casa e a scuola)? (Cerchia una risposta)

1. Per Nulla 2. Un po’ 3. Moderatamente 4. Un bel po’ 5. Estremamente

Quali aspettative ha per il trattamento di Suo/a figlio/a?
Come risultato del trattamento, mi aspetto che mio/a figlio/a: (Cerchia una risposta su ogni riga.)

		**Sicuramente si**	**Probabilmente si**	**Non sono sicuro**	**Probabilmente no**	**Sicuramente no**
**77.**	Abbia meno dolore	1	2	3	4	5
**78.**	Abbia un aspetto migliore	1	2	3	4	5
**79.**	Si senta meglio con sé stesso	1	2	3	4	5
**80.**	Dorma più comodamente	1	2	3	4	5
**81.**	Sia in grado di svolgere più attività in casa	1	2	3	4	5
**82.**	Sia in grado di poter fare di più a scuola	1	2	3	4	5
**83.**	Sia in grado di giocare di più con gli amici.	1	2	3	4	5
**84.**	Sia in grado di praticare più sport	1	2	3	4	5
**85.**	Non abbia dolore e limitazione da adulto	1	2	3	4	5

86. Se Suo/a figlio/a dovesse passare il resto della tua vita con le sue condizioni ossee e muscolari **come sono adesso**, come si sentirebbe al riguardo? (Cerchia una risposta)

1. Molto soddisfatto
2. Più o meno soddisfatto
3. Né soddisfatto, né insoddisfatto
4. Più o meno insoddisfatto
5. Molto insoddisfatto

Questionario per

Bambini

Creato da:

American Academy of Orthopaedic Surgeons®

Pediatric Orthopaedic Society of North America

American Academy of Pediatrics

Shriner's Hospitals

Deve essere compilato dai genitori del bambino tra i 2 – 10 anni

Basato sulla Versione 2.0 del Pediatrics–Parent/ Adolescent Outcomes Intrument

Conosciuto anche come PODCI ("Pediatric Outcomes Data Collection Instrument")

Valutazione della Salute dei Bambini (compilato dai genitori)

Data di oggi / /

Grazie per aver completato questo questionario!

Questo questionario ci aiuterà a comprendere meglio lo stato generale di salute di Suo/a figlio e gli eventuali problemi legati alle condizioni ossee e muscolari.

La compilazione di questo questionario è completamente volontaria e le Sue risposte saranno mantenute con la massima riservatezza.

Per favore risponda ad ogni domanda. Alcune domande possono assomigliare ad altre, ma ognuna è diversa.

Non ci sono risposte giuste o sbagliate. Se non è sicuro di come rispondere a una domanda, dia solo la migliore risposta possibile. Può inserire commenti a margine. Leggeremo tutti i tuoi commenti, quindi sentiti libero di farne quanti ne desideri.

Data di nascita del paziente / /

Valutazione della Salute dei Bambini (compilato dai genitori)

Alcuni problemi possono rendere difficile lo svolgimento di molte attività, come mangiare, fare il bagno, fare i compiti scolastici e giocare con gli amici. Vorremmo scoprire come sta Suo/a/a figlio/a.
(Cerchia una risposta su ogni riga.)

Durante **l'ultima settimana** quanto è stato difficile per Suo/a figlio/a:

		Facile	Un po' difficile	Molto difficile	Non ci sono riuscito
**1.**	Sollevare libri pesanti?	1	2	3	4
**2.**	Versare un litro di latte?	1	2	3	4
**3.**	Aprire un barattolo precedentemente aperto?	1	2	3	4
**4.**	Usare una forchetta o un cucchiaio?	1	2	3	4
**5.**	Pettinarsi?	1	2	3	4
**6.**	Abbottonare una camicia?	1	2	3	4
**7.**	Indossare una giacca?	1	2	3	4
**8.**	Scrivere con una penna?	1	2	3	4

9. In media, **negli ultimi 12 mesi**, quanto spesso Suo/a figlio/a ha saltato la scuola per motivi di salute?

1. Raramente
2. Una volta al mese
3. Due o tre volte al mese
4. Una volta a settimana
5. Più di una volta a settimana
6. Non va a scuola

Durante **l'ultima settimana** quanto Suo/a figlio/a si è sentito a tuo agio con: (Cerchia una risposta per ogni domanda.)

		Molto felice	Un po' felice	Insicuro	Un po' triste	Molto infelice
**10.**	Il Suo aspetto?	1	2	3	4	5
**11.**	Il Suo corpo?	1	2	3	4	5
**12.**	I vestiti e le scarpe che indossa?	1	2	3	4	5
**13.**	La Sua capacità di fare le stesse cose che fanno i Suoi amici?	1	2	3	4	5
**14.**	La Sua salute in generale?	1	2	3	4	5

Durante **l'ultima settimana**, quanto tempo: (cerchia una risposta ad ogni domanda)

		La maggior parte delle volte	Qualche volta	Un po' di volte	Mai
**15.**	Suo/a figlio/a si è sentito stanco?	1	2	3	4
**16.**	Suo/a figlio/a si è sentito pieno di energia?	1	2	3	4
**17.**	Il dolore ha interferito con le attività di Suo/a figlio/a?	1	2	3	4

Durante **l'ultima settimana**, è stato facile o difficile per Suo/a figlio/a: (cerchia una risposta ad ogni domanda)

		Facile	Un po' difficile	Molto difficile	Non ci sono riuscito
**18.**	Correre per brevi distanze?	1	2	3	4
**19.**	Andare in bicicletta?	1	2	3	4
**20.**	Salire tre rampe di scale?	1	2	3	4
**21.**	Salire una rampa di scale?	1	2	3	4
**22.**	Camminare per più di un chilometro?	1	2	3	4
**23.**	Camminare per tre isolati?	1	2	3	4
**24.**	Camminare per un isolato?	1	2	3	4
**25.**	Salire e scendere dall'autobus?	1	2	3	4

26. Con che frequenza Suo/a figlio/a ha bisogno dell'aiuto di un'altra persona per camminare e/o arrampicarsi? (Cerchia una risposta)

1 Mai   2 A volte   3 Circa la metà delle volte   4 Spesso   5 Sempre

27. Con che frequenza Suo/a figlio/a usa dispositivi di assistenza (tutori, stampelle o sedia a rotelle) per camminare e/o arrampicarsi? (Cerchia una risposta)

1 Mai   2 A volte   3 Circa la metà delle volte   4 Spesso   5 Sempre

Durante **l'ultima settimana**, quanto è stato facile per Suo/a figlio/a:(cerchia una risposta ad ogni domanda)

		Facile	Un po' difficile	Molto difficile	Non ci sono riuscito
**28.**	Stare in piedi per lavarsi le mani e la faccia?	1	2	3	4
**29.**	Sedersi su una sedia normale senza tenersi?	1	2	3	4
**30.**	Salire e scendere dal gabinetto?	1	2	3	4
**31.**	Salire e scendere dal letto?	1	2	3	4
**32.**	Aprire la maniglia della porta?	1	2	3	4
**33.**	Piegarsi in avanti per raccogliere qualcosa da terra?	1	2	3	4

34. Con che frequenza Suo/a figlio/a ha bisogno dell'aiuto di un'altra persona per stare seduto o in piedi? (Cerchia una risposta)

1 Mai   2 A volte   3 Circa la metà delle volte   4 Spesso   5 Sempre

35. Con che frequenza Suo/a figlio/a utilizza dispositivi di assistenza (come tutori, stampelle o sedia a rotelle) per stare seduto o in piedi? (Cerchia una risposta)

1 Mai   2 A volte   3 Circa la metà delle volte   4 Spesso   5 Sempre

36. Riesce Suo/a figlio/a a partecipare ad **attività ricreative all'aperto** con altri bambini della sua stessa età? Ad esempio: ciclismo, pattinaggio, escursionismo, jogging... (Cerchia una risposta).

1 Si, facilmente   2 Si, ma è un po' difficile   3 Si, ma è molto difficile   4 No

Se ha risposto "no" alla domanda 36, l'attività di Suo/a figlio/a era limitata da: (Cerchia sì a tutte le risposte pertinenti)

	Si
**37.** Dolore?	1
**38.** Il Suo livello di salute generale?	1
**39.** Indicazioni del Medico o dei Genitori?	1
**40.** Ha paura che Suo/a figlio/a non piaccia agli altri?	1
**41.** A Suo/a figlio/a non piacciono gli sport all'aperto?	1
**42.** Reputa Suo/a figlio/a troppo piccolo?	1
**43.** Attività fuori stagione?	1

44. Riesce Suo/a figlio/a a partecipare in **giochi o sport di squadra** con altri ragazzi della sua stessa età? (Ad esempio: basket, calcio, pallavolo, salto con la fune…) (Cerchia una risposta).

1 Si, facilmente   2 Si, ma è un po' difficile   3 Si, ma è molto difficile   4 No

Se ha risposto "no" alla domanda 43, l'attività di Suo/a figlio/a era limitata da: (Cerchia tutte le risposte pertinenti)

	Si
**45.** Dolore?	1
**46.** Il Suo/a livello di salute generale?	1
**47.** Indicazioni del Medico o dei Genitori?	1
**48.** Ha paura che Suo/a figlio/a non piaccia agli altri?	1
**49.** A Suo/a figlio/a non piacciono gli sport di squadra?	1
**50.** Reputa Suo/a figlio/a troppo piccolo?	1
**51.** Attività fuori stagione?	1

Valutazione della Salute degli Adolescenti (compilato dai genitori)

52. Riesce Suo/a figlio/a a partecipare **a sport di livello agonistico** con altri ragazzi della sua stessa età? (Ad esempio: hockey, basket, calcio, baseball, nuoto, corsa [pista o sci di fondo], ginnastica o danza) (Cerchia una risposta).

1 Si, facilmente   2 Si, ma è un po' difficile   3 Si, ma è molto difficile   4 No

Se ha risposto "no" alla domanda 50, la Sua attività era limitata da: (Cerchia tutte le risposte pertinenti)

	Si
**53.** Dolore?	1
**54.** Il Suo livello di salute generale?	1
**55.** Indicazioni del Medico o dei Genitori?	1
**56.** Ha paura che Suo/a figlio/a non piaccia agli altri?	1
**57.** A Suo/a figlio/a non piacciono gli sport a livello agonistico?	1
**58.** Reputa Suo/a figlio/a troppo piccolo?	1
**59.** Attività fuori stagione?	1

60. Quante volte **<u>nell'ultima settimana</u>** Suo/a figlio/a si è incontrato con i Suoi amici per fare attività? (cerchia una risposta)

1 Spesso   2 A volte   3 Raramente o Mai

Se ha risposto "A volte" o "Raramente o Mai" alla domanda 57, l'attività di Suo/a figlio/a era limitata da: (Cerchia tutte le risposte pertinenti)

	Si
**61.** Dolore?	1
**62.** Il Suo livello di salute generale?	1
**63.** Indicazioni del Medico o dei Genitori?	1
**64.** Ha paura che Suo/a figlio/a non piaccia agli altri?	1
**65.** Gli amici non erano disponibili?	1

66. Quante volte **<u>nell'ultima settimana</u>** Suo/a figlio/a ha partecipato alla **ginnastica scolastica/ricreazione**?

1 Spesso   2 A Volte   3 Mai o Raramente   4 Non abbiamo ore di ginnastica/ricreazione

Se ha risposto "A Volte" o "Mai o Raramente" alla domanda 66, l'attività di Suo/a figlio/a era limitata da: (Cerchia tutte le risposte pertinenti)

	Si
**67.** Dolore?	1
**68.** Il Suo livello di salute generale?	1
**69.** Indicazioni del Medico o dei Genitori?	1
**70.** Ha paura che Suo/a figlio/a non piaccia agli altri?	1
**71.** A Suo/a figlio/a non ti piace fare ginnastica a scuola?	1
**72.** Non siamo in periodo scolastico?	1
**73.** Suo/a figlio/a non frequenta la scuola	1

74. È facile o difficile per Suo/a figlio/a fare amicizia con ragazzi della sua età? (Cerchia una risposta)

1 Generalmente Facile   2 Spesso Facile   3 Spesso Difficile   4 Generalmente Difficile

75. Quanto dolore ha avuto Suo/a figlio/a durante **l'ultima settimana**? (Cerchia una risposta)

1. Nessuno   2. Molto Poco   3. Lieve   4. Moderato   5. Severo   6. Molto severo

76. Durante **l'ultima settimana**, quanto il dolore ha interferito con le normali attività di Suo/a figlio/a (comprese quelle a casa, fuori casa e a scuola)? (Cerchia una risposta)

1. Per Nulla   2. Un po'   3. Moderatamente   4. Un bel po'   5. Estremamente

Quali aspettative ha per il trattamento di Suo/a figlio/a?
Come risultato del trattamento, mi aspetto che mio/a figlio/a: (Cerchia una risposta su ogni riga.)

		Sicuramente si	Probabilmente si	Non sono sicuro	Probabilmente no	Sicuramente no
**77.**	Abbia meno dolore	1	2	3	4	5
**78.**	Abbia un aspetto migliore	1	2	3	4	5
**79.**	Si senta meglio con sé stesso	1	2	3	4	5
**80.**	Dorma più comodamente	1	2	3	4	5
**81.**	Sia in grado di svolgere più attività in casa	1	2	3	4	5
**82.**	Sia in grado di poter fare di più a scuola	1	2	3	4	5
**83.**	Sia in grado di giocare di più con gli amici.	1	2	3	4	5
**84.**	Sia in grado di praticare più sport	1	2	3	4	5
**85.**	Non abbia dolore e limitazione da adulto	1	2	3	4	5

86. Se Suo/a figlio/a dovesse passare il resto della tua vita con le sue condizioni ossee e muscolari **come sono adesso**, come si sentirebbe al riguardo? (Cerchia una risposta)

1. Molto soddisfatto
2. Più o meno soddisfatto
3. Né soddisfatto, né insoddisfatto
4. Più o meno insoddisfatto
5. Molto insoddisfatto
