# Supplementary material for: Translation and Cross-Cultural Adaptation of the Pediatric Outcomes Data Collection Instrument into the Italian Language
Source: Children (Basel). 2022 Jun 8;9(6):853. doi: 10.3390/children9060853 (PMC9221952; doi:10.3390/children9060853)
Supplement: Supplementary file 1 [file children-09-00853-s001.zip › Supplementary Table S1AB.pdf]

Supplementary Table S1. Spearman rho correlation of age with Psychometric properties of Pediatric Outcome Data Collection Instrument (PODCI) Adolescent self-reported form (N=39)

PODCI Subscale	T0[ǂ]		T2[†]	
	rho	*p*-value[*]	rho	*p*-value
Upper Extremity Function	.29	.08	.06	.70
Transfers & Basic Mobility	-.16	.32	-.28	.08
Sport/Physical Functioning	-.17	.30	-.08	.63
Pain/Comfort	-.15	.36	-.24	.14
Happiness	-.08	.65	.04	.79
Expectations	.02	.90	.015	.93
Global Functioning	-.14	.38	-.19	.24

[*] $p < 0.05$
[ǂ] T0: baseline measurement; [†] T2: 3-month surgical treatment follow-up measurment

Supplementary Table S2. Spearman rho correlation of age with Psychometric properties of Pediatric Outcome Data Collection Instrument (PODCI) Parent-reported form (N=20)

PODCI Subscale	T0[ǂ]		T2[†]	
	rho	*p*-value[*]	rho	*p*-value
Upper Extremity Function	.69	.001	.175	.46
Transfers & Basic Mobility	.43	.06	.46	.04
Sport/Physical Functioning	.32	.17	.34	.14
Pain/Comfort	.53	.01	-.01	.97
Happiness	-.07	.76	-.01	.95
Expectations	.61	.004	.26	.26
Global Functioning	-.36	.12	.002	.99

[*] $p < 0.05$
[ǂ] T0: baseline measurement; [†] T2: 3-month surgical treatment follow-up measurment
